# Supplementary material for: Atmospheric turbulence strength distribution along a propagation path probed by longitudinally structured optical beams
Source: Nat Commun. 2023 Aug 5;14:4701. doi: 10.1038/s41467-023-40381-z (PMC10404261; doi:10.1038/s41467-023-40381-z)
Supplement: Supplementary file 1 — Supplementary Information [file 41467_2023_40381_MOESM1_ESM.pdf]

Supplementary Information: Atmospheric Turbulence Strength Distribution along a Propagation Path Probed by Longitudinally Structured Optical Beams

Huibin Zhou[1,*], Xinzhou Su[1], Yuxiang Duan[1], Hao Song[1], Kaiheng Zou[1], Runzhou Zhang[1], Haoqian Song[1], Nanzhe Hu[1], Moshe Tur[2], and Alan E. Willner[1,3,*]

[1]*Department of Electrical and Computer Engineering, University of Southern California, Los Angeles, CA 90089, USA.*

[2]*School of Electrical Engineering, Tel Aviv University, Ramat Aviv 69978, Israel*

[3]*Dornsife Department of Physics & Astronomy, University of Southern California, Los Angeles, CA 90089, USA*

**Correspondence to: huibinzh@usc.edu, willner@usc.edu.*

The supplementary materials are organized as follows: In Section 1, we show simulations and calculations to examine the use of Eqs. (1-2) in the main text for our inhomogeneous case. In Section 2, we perform simulations to investigate the effects of turbulence-induced beam width variations on our approach. In Section 3, we provide additional simulation results of our approach for probing various longitudinal distributions of turbulence strength. In Section 4, we show the simulation for comparing the focused Gaussian beams and the longitudinally structured beams in terms of their beam width changes along z. In Section 5, we describe the method of modal spectrum measurement using off-axis holography used in our experiment. In Section 6, we experimentally examine our emulated turbulence by measuring the Strehl ratio and the power fluctuation for a Gaussian beam.

Supplementary Note 1. Simulations and calculations to examine the use of Eqs. (1-2) in the main text for inhomogeneous turbulence cases

To help examine the use of Eqs. (1-2) in the main text for inhomogeneous turbulence scenarios, we simulate beam propagation through different turbulence distributions each comprising three turbulence regions. As shown in Fig. S1, we simulate $P_{i,j}(\ell = 0)$ for beam i at the output plane of region j using spatial modal decomposition (see "*Methods*" in the main text). We also calculate $P_{i,j}(\ell = 0)$ based on Eqs. (1-2) and compare the results to the simulated ones. The calculation methods are as follows:

- Calculation based on Eqs. (1-2):

Eqs. (1-2) describe how much power will be coupled from the $\ell = 0$ order to other $\ell \neq 0$ orders due to turbulence-induced modal coupling [24]. Based on Eqs. (1-2), we can calculate the normalized average power remaining on the $\ell = 0$ order ($P(\ell = 0)$) after the beam propagates through a turbulence region. In inhomogeneous turbulence cases with multiple uniform turbulence regions, we define $P_{i,j}(\ell = 0)$ as the power remaining on the $\ell = 0$ order for beam i at the end of region j. Due to the accumulated effects of multiple turbulence regions, there can be increased modal power coupling during beam propagation and the resulting $P_{i,j}(\ell = 0)$ will decrease with a larger j.

For example, after propagation through the first turbulence region, $P_{i,1}(\ell = 0)$ can be calculated based on Eqs. (1-2) [24], as follows:

$$P_{i,1}(\ell = 0) = \left(I_0(\beta_{i,1}) + I_1(\beta_{i,1})\right) exp(-\beta_{i,1}) \tag{S1}$$

and

$$\beta_{i,1} \approx 1.8025(D_{i,1})^{\frac{5}{3}}[0.423k^2 C_{n,1}^2 \Delta z] \tag{S2}$$

where $D_{i,1}$ is the beam width of beam i in the first region, and $C_{n,1}^2$ is the turbulence strength of the first region.

After propagating through the next turbulence regions, the beam experiences stronger modal coupling, and more power on the $\ell = 0$ order will be coupled to $\ell \neq 0$ orders. As a result, the relative power remaining on the $\ell = 0$ will decrease and the $P_{i,j}(\ell = 0)$ is calculated as follows [24]:

$$P_{i,j}(\ell = 0) \approx P_{i,0}(\ell = 0) \prod_{m=1}^{j} \left(I_0(\beta_{i,m}) + I_1(\beta_{i,m})\right) exp(-\beta_{i,m}) \tag{S3}$$

and

$$\beta_{i,m} \approx 1.8025(D_{i,m})^{\frac{5}{3}}[0.423k^2 C_{n,m}^2 \Delta z] \tag{S4}$$

where the value of $P_{i,0}(\ell = 0)$ is 1 at the transmitter, $D_{i,m}$ is the beam width in region m, and $C_{n,m}^2$ is the turbulence strength of region m.

- Simulation and calculation results:

As shown in Fig. S1, our simulation and calculation results show that $P(\ell = 0)$ becomes smaller after the beam propagates through more turbulence regions. This might be because larger accumulated turbulence effects cause more power to be coupled from the $\ell = 0$ order. Moreover, a stronger turbulence region causes a greater decrease in the $P(\ell = 0)$ for each beam due to the stronger modal coupling effect in this region. The calculated results are in relative agreement with the simulated results and show <5% average relative errors.

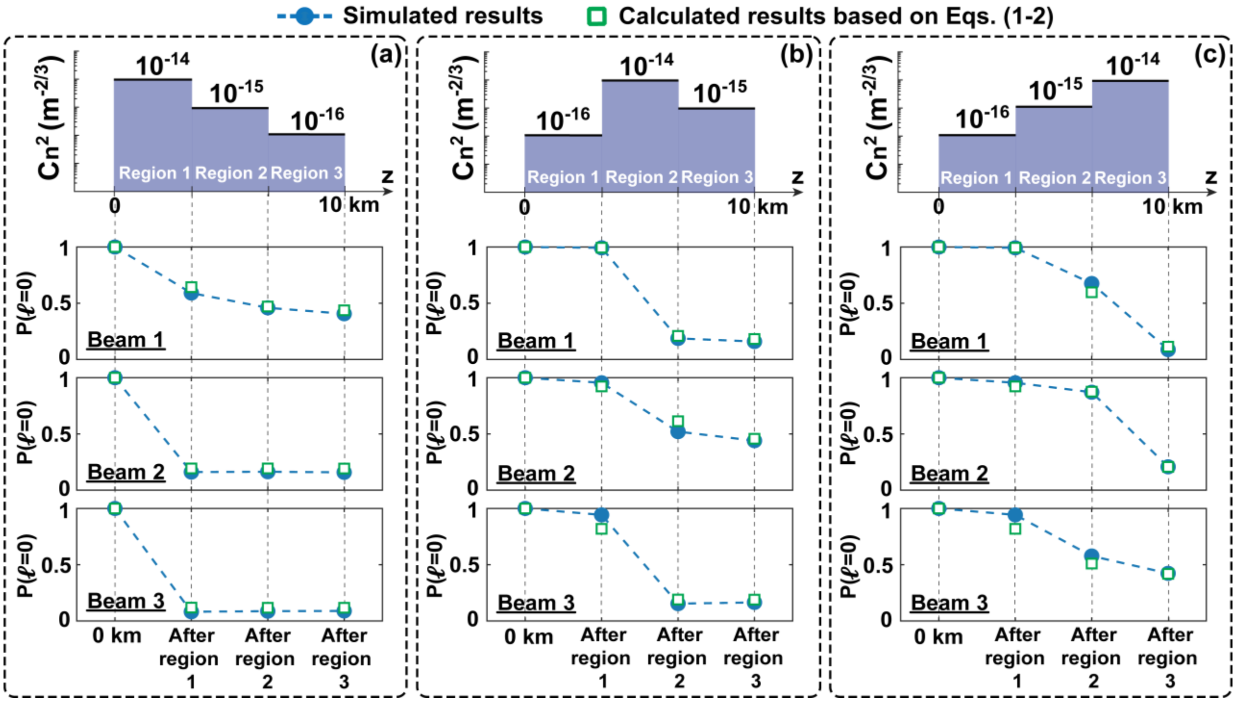

Fig. S1. Simulated and calculated $P(\ell = 0)$ values for each probe beam under three different turbulence strength distribution cases each containing three turbulence regions. For each case, $P(\ell = 0)$ values are simulated and calculated at the end of each region.

Supplementary Note 2. Simulations to investigate the effects of turbulence-induced beam width variations on our approach

We simulate the turbulence-induced beam width variation and its effects on our probing approach. As shown in Fig. S2, we simulate different turbulence distribution cases each containing three turbulence regions. We design three sequentially transmitted probe beams to probe these distributions along a 10-km path. For each probe beam, we first simulate its propagation in the vacuum (without turbulence) and calculate its beam width at various distances (see "*Methods*" in the main text for beam width calculation). We can find that the beam width is smaller in one specific region (i.e., smaller-beam-width region) as we designed.

Next, we simulate beam propagation through different turbulence distributions and calculate the beam width at different distances under 200 turbulence realizations. The orange shades in Fig. S2 show the range of beam width variations induced by turbulence, which is larger for a longer propagation distance due to stronger accumulated turbulence effects [1,2]. When the stronger turbulence region is closer to the transmitter, the beam width has larger variations (e.g., comparing Case 1 to Case 3). This might be due to that severe turbulence distortion near the transmitter causes stronger beam variations after a longer-distance

propagation [3]. We also simulate the average beam width in turbulence and compare it to the designed beam width in the vacuum. The results show that the average beam width in turbulence is larger than that in the vacuum due to turbulence-induced beam spreading [4].

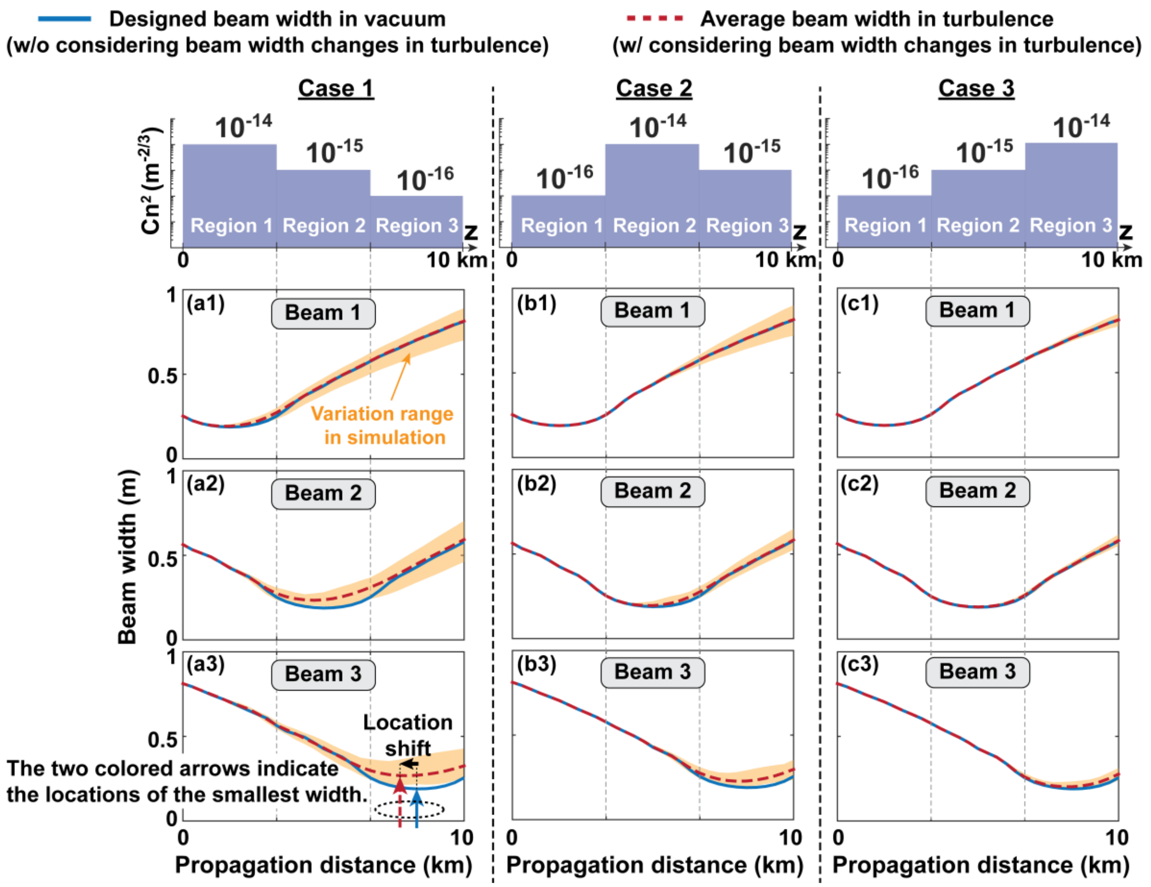

Fig. S2. Simulated beam width for each probe beam with three different turbulence distributions. The designed beam width in the vacuum without turbulence is also simulated for comparison. The orange shades show the range of beam width variations under 200 turbulence realizations.

Besides the beam spreading, turbulence-induced beam width variations can also change the longitudinal location of the smaller-beam-width region [5,6]. An example is indicated in Fig. S2 (a3), where the location of the smaller-beam-width region will be shifted closer to the transmitter under turbulence. Previous studies have also shown a similar effect for focused Gaussian beams, in which the beam waist location shifts closer to the transmitter under turbulence [5,6].

We subsequently simulate how the turbulence-induced beam width variations affect our probing approach. Figure. S3 (a) shows an original turbulence distribution. Figure. S3 (b) shows simulated and theoretically calculated $P(\ell = 0)$ values for each probe beam at the receiver. We calculate $P(\ell = 0)$ using

the designed beam width in the vacuum and the average beam width in turbulence. Compared to the designed beam width in the vacuum, the calculation results using the average beam width in turbulence are slightly closer (~3%) to the simulation results on average. This might be because (i) the average beam width in turbulence is larger, corresponding to more turbulence-induced modal coupling, and (ii) the designed beam width in the vacuum might underestimate the modal coupling. Subsequently, we use the beam width in the vacuum or the average beam width in turbulence to form equations for retrieving turbulence. As shown in Fig. S3 (c), the probing error is ~2% smaller when using the average beam width in turbulence.

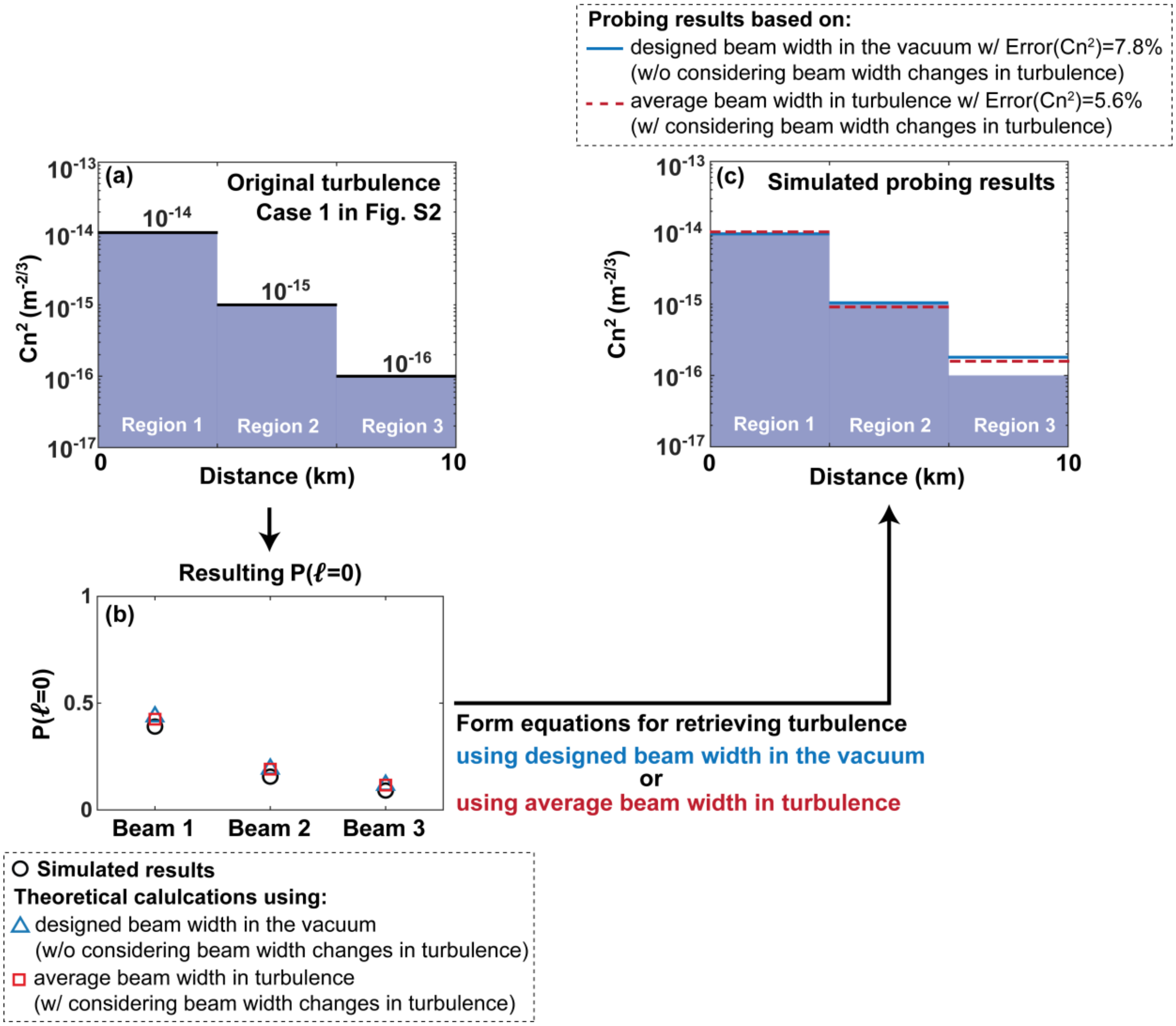

Fig. S3. Simulated $P(\ell = 0)$ and turbulence probing results with and without considering turbulence-induced beamwidth changes. (a) Original turbulence distribution in simulation. (b) Simulated and theoretically calculated $P(\ell = 0)$ for each probe beam. (c) Simulated probing results when using the designed beam width in the vacuum and the average beam width in turbulence to form equations for retrieving turbulence.

Supplementary Note 3. Simulated performance of our approach for probing various turbulence distributions

In order to show the feasibility of our approach under more general scenarios, we simulate additional turbulence distributions with different shapes, as shown in Fig. S4. In this simulation, we set $Q = (1 - 6 \times 10^{-10}) \times k$ and $N = 7$. These distributions include linearly changing distributions (Fig. S4 (a-b)), "triangular-shaped" distributions (Fig. S4 (c-d)), and "sine-shaped" distributions (Fig. S4 (e-f)). Figure. S4 (g) shows the simulation results for an atmospheric turbulence profile based on the Hufnagel-Valley (H-V) model [7]. We also simulate our approach and compare it to experimental measurements of C_n^2 by radiometers at different altitudes in the literature [8], as shown in Fig. S5 (h). Our results show that (i) the probed turbulence distribution over altitudes has a similar trend as the original turbulence and (ii) the simulated probing error compared to the H-V model and the experimental data in the literature is ~8% and ~16%, respectively. From the calculated relative average probing errors for these turbulence distributions, more complicated distributions tend to result in larger errors, which might be due to the larger longitudinally spatial gradients, characteristic of these simulated distributions.

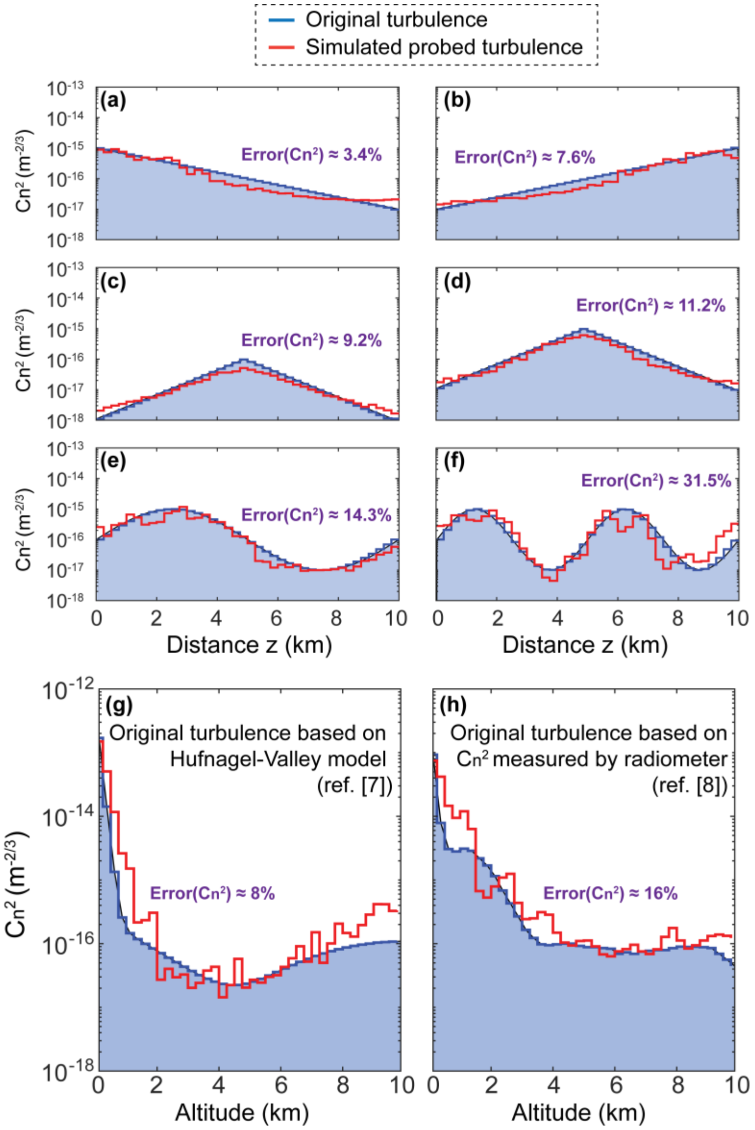

Fig. S4. Simulated turbulence probing results for different turbulence strength distributions. (a) Simulation results for probing turbulence with various distributions, including (a-b) linear changing distributions, (c-d) "triangular-shaped" distributions, (e-f) "sin-shaped" distributions, and (g) distribution based on the Hufnagel-Valley model describing the atmospheric turbulence distribution at different altitudes, and (h) experimentally measured C_n^2 at different altitudes by radiometers.

Supplementary Note 4. Simulation for comparing the focused Gaussian beams and the longitudinally structured beams in terms of the beam width changes along z

To compare the focused single Gaussian beam approach [9,10] with the longitudinally structured beam of this paper, we simulate the propagation of a Gaussian beam focused by different lenses with the focal point located at z=0.1, 1, 5, and 9 km and compare it to a longitudinally structured beam with its probe region

tuned to the same distances of z=0.1, 1, 5, and 9 km in a 10-km free-space link. The longitudinally structured beam is generated by a superposition of Bessel-Gauss modes, apodized by a Gaussian aperture [11], which has the same width as the focused Gaussian beam at the Tx (z=0). In this simulation, we set $Q = (1 - 6 \times 10^{-10}) \times k$ and $N = 7$ for the longitudinally structured beam. Figures S5 (a) and (b) show the simulated beam width for the focused Gaussian beam and longitudinally structured beam, respectively. Compared to the longitudinally structured beam, the focused Gaussian beam has shaper beam width changes especially when the focal point is near to the Tx. This behavior might provide a finer longitudinally spatial resolution for turbulence probing as we discussed in the *"Simulation"* section of the main text. However, after the focal point, the focused Gaussian beam diverges faster than the longitudinally structured beam, resulting in larger beam widths at the Rx (z=10 km). When the focal point is at z=100 m and z=1 km, the focused Gaussian beam has ~8X and ~3X larger beam width than the longitudinally structured beam. In Fig. S5 (c), we also simulate the power loss induced by an Rx aperture with a 1-m diameter for these two beams. The focused Gaussian beam suffers a larger power loss when the focal point is near the Tx.

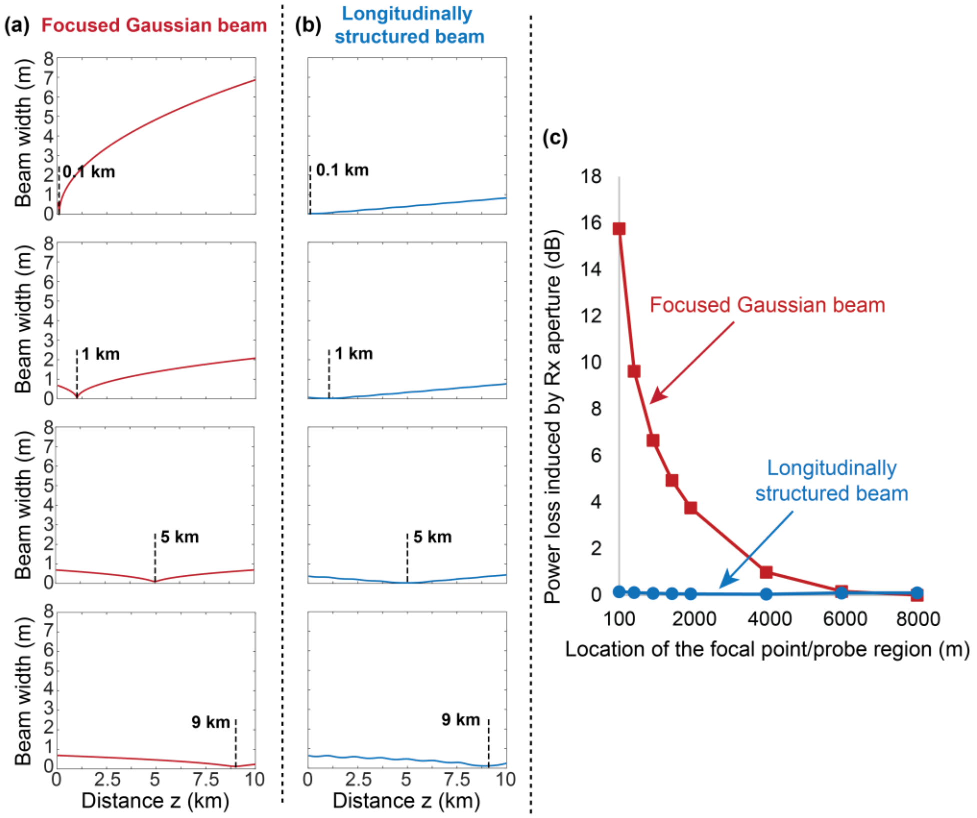

Fig. S5. Simulated beam width and power loss caused by a limited-size Rx aperture along z in a 10-km link. (a) A focused Gaussian beam and (b) a longitudinally structured beam when the focal point/probe region is located at z=0.1, 1, 5, and 9 km. (c) Simulated power loss caused by a limited-size Rx aperture with a 1-m diameter for these two beams when the focal point/probe region is at a different z.

Supplementary Note 5. Modal spectrum measurement using off-axis holography

In the Experimental Validation section of the main text, we show the spatial intensity and phase profiles and measured OAM spectra of the turbulence-distorted longitudinally structured beam. Here we present the steps we performed to extract the complex beam profiles of a distorted beam using off-axis holography [12]. As shown in Fig. S6, we perform the following steps:

Step 1: Using an infrared camera to record the interferogram between the distorted beam and another off-axis undistorted coherent reference Gaussian beam.

Step 2: Calculating the spatial frequency spectrum of the interferogram through the two-dimensional Fourier transform.

Step 3: Filtering out the 1st-order diffraction and shifting it to the center of the spatial frequency spectrum.

Step 4: Converting the shifted spatial frequency spectrum to the spatial space by the two-dimensional inverse Fourier transform to obtain the spatial amplitude and phase profiles of the distorted beam.

Step 5: Decompose the distorted beam into the Bessel modal basis and calculate the OAM modal spectrum using the method described in *Method* section of the main text.

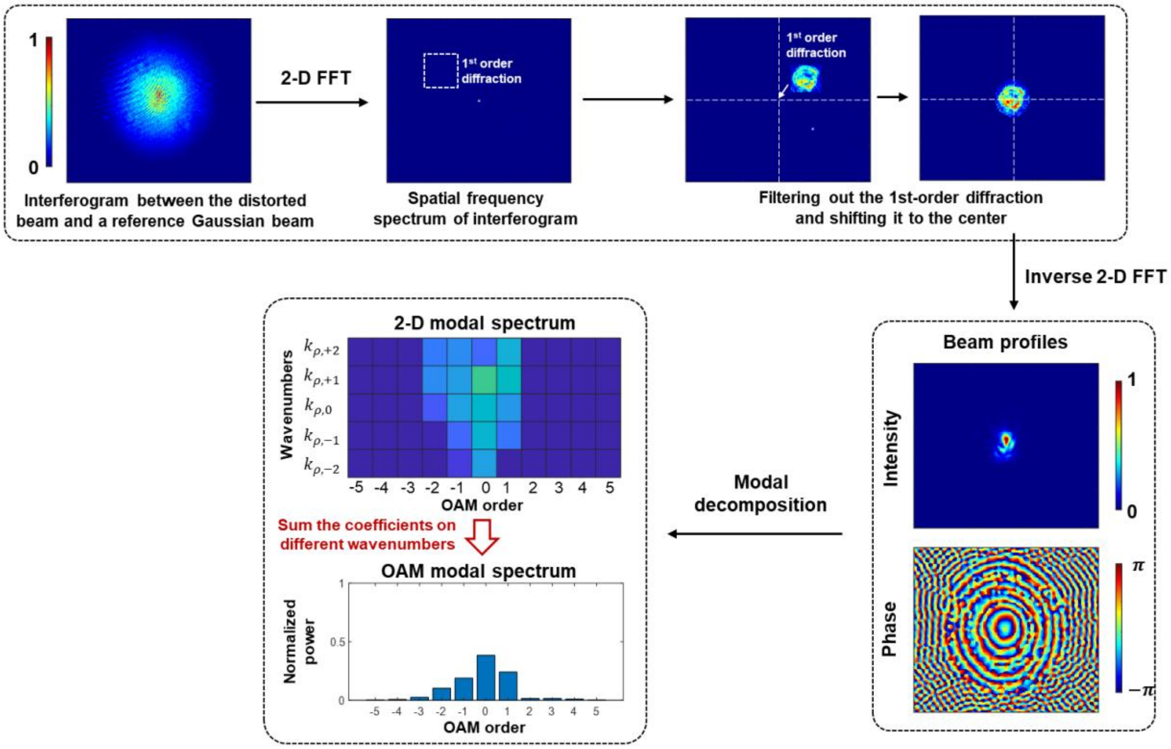

Fig. S6. Off-axis holography approach to extract the complex beam profiles of a distorted longitudinally structured beam. The interferogram is recorded by an infrared camera with 320x256 pixels (the size of each pixel is 30μm).

Supplementary Note 6. Measured Strehl ratio and power fluctuation for a Gaussian beam propagating through a turbulence phase plate

As shown in Fig. S7 (a), we measure the Strehl ratio (SR) [1] for different phase plates (different r_0) with various path lengths (i.e., L= 0.3 and 0.6 m). To measure each data point, we place the corresponding phase plate in the middle of the path (i.e., z = L/2) and propagate a Gaussian beam through it. The width of the Gaussian beam is D=3.5 mm. At the receiver, we measure SR values and compare them to the theoretical ones, which can be expressed as $[1+(D/r_0)^{5/3}]^{(-6/5)}$ [1]. Our results show that the measured values are close to the theoretical ones with <8% relative errors. In Fig. S7 (b), we measure the received power fluctuation of the Gaussian beam for the phase plate with $r_0 = 1$ mm. For these measurements, the receiver aperture diameter is ~1 mm. Our results show that the probability density function of measured power fluctuations follows a lognormal model for each path length. The correlation coefficient R between the distribution and its lognormal fitting curve is >0.96. We also calculate the scintillation index σ_I^2 [1] and find that it increases from 0.093 to 0.161 when L changes from 0.3 to 0.6 m.

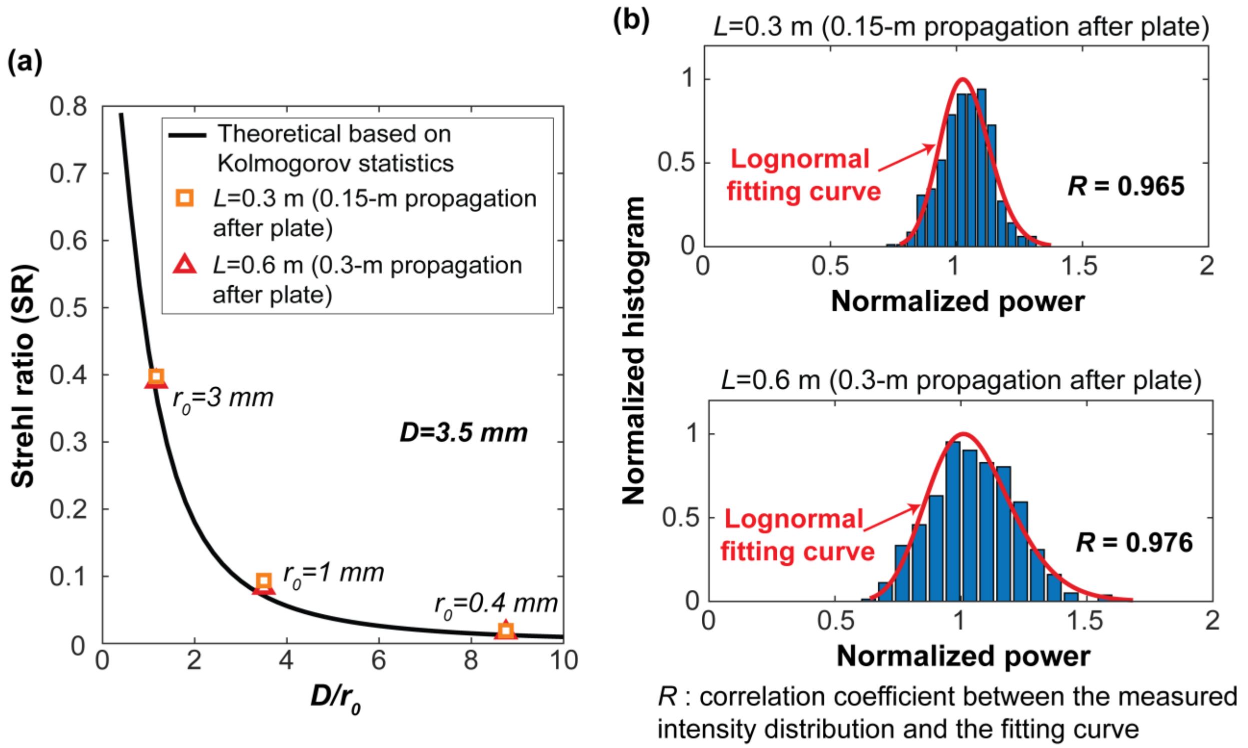

Fig. S7. Measured Strehl ratio and power fluctuation for a Gaussian beam propagating through a turbulence phase plate. (a) Measured SR for different phase plates (different r_0) with different path lengths (different L). The beam width of the Gaussian beam is D=3.5 mm. (b) Measured probability density function of power fluctuations of the received beam for the phase plate with $r_0 = 1$ mm.

Reference for the supplementary information:

1. L. C. Andrews and R. L. Phillips, "Laser beam propagation through random media," Laser Beam Propagation Through Random Media: Second Edition (2005).
2. H. T. Yura, "Short-term average optical-beam spread in a turbulent medium," J. Opt. Soc. Am. 63, 567-572 (1973).
3. Y. K. Chahine, S. A. Tedder, B. E. Vyhnalek, and A. C. Wroblewski. "Beam propagation through atmospheric turbulence using an altitude-dependent structure profile with non-uniformly distributed phase screens," In Free-Space Laser Communications XXXII, vol. 11272, pp. 263-277. SPIE, 2020.
4. C. Y. Young, Y. V. Gilchrest, and B. R. Macon. "Turbulence induced beam spreading of higher order mode optical waves," Optical Engineering 41, 1097-1103 (2002).
5. J. C. Ricklin, W. B. Miller, and L. C. Andrews. "Optical turbulence effects on focused laser beams: new results," In Optics in Atmospheric Propagation and Random Phenomena, vol. 2312, pp. 145-154. SPIE, 1994.
6. J. C. Ricklin, W. B. Miller, and L. C. Andrews. "Effective beam parameters and the turbulent beam waist for convergent Gaussian beams," Applied Optics 34, 7059-7065 (1995).
7. L. C. Andrews, Field Guide to Atmospheric Optics (SPIE, 2004).
8. Brian E. Vyhnalek, "Path profiles of Cn2 derived from radiometer temperature measurements and geometrical ray tracing," In Free-Space Laser Communication and Atmospheric Propagation XXIX, vol. 10096, pp. 386-396. SPIE, 2017.
9. A. Ishimaru, "The beam wave case and remote sensing," in Laser Beam Propagation in the Atmosphere, J. W. Strohbehn, ed., Topics in Applied Physics (Springer Berlin Heidelberg, 1978), vol. 25, pp. 129–170.
10. A. Ishimaru, "Fluctuations of a focused beam wave for atmospheric turbulence probing," Proc. IEEE 57, 407–414 (1969).
11. M. Zamboni-Rached and M. Mojahedi, "Shaping finite-energy diffraction- and attenuation-resistant beams through Bessel-Gauss–beam superposition," Phys. Rev. A 92, 043839 (2015).
12. R. Zhang, N. Hu, H. Zhou, K. Zou, X. Su, Y. Zhou, H. Song, K. Pang, H. Song, A. Minoofar, Z. Zhao, C. Liu, K. Manukyan, A. Almaiman, B. Lynn, R. W. Boyd, M. Tur, and A. E. Willner, "Turbulence-resilient pilot-assisted self-coherent free-space optical communications using automatic optoelectronic mixing of many modes," Nat. Photon. 15, 743–750 (2021).
