# Supplementary material for: Atmospheric turbulence strength distribution along a propagation path probed by longitudinally structured optical beams
Source: Nat Commun. 2023 Aug 5;14:4701. doi: 10.1038/s41467-023-40381-z (PMC10404261; doi:10.1038/s41467-023-40381-z)
Supplement: Supplementary file 3 — Reporting Summary [file 41467_2023_40381_MOESM3_ESM.pdf]

nature research

Corresponding author(s): Huibin Zhou

# Lasing Reporting Summary

Nature Research wishes to improve the reproducibility of the work that we publish. This form is intended for publication with all accepted papers reporting claims of lasing and provides structure for consistency and transparency in reporting. Some list items might not apply to an individual manuscript, but all fields must be completed for clarity.

For further information on Nature Research policies, including our data availability policy, see Authors & Referees.

## Experimental design

**Please check: are the following details reported in the manuscript?**

1. Threshold

Plots of device output power versus pump power over a wide range of values indicating a clear threshold

☐ Yes ☒ No

Our work studies the spatial structure properties of optical beams, Therefore, it is not relevant to the threshold of the laseing.

2. Linewidth narrowing

Plots of spectral power density for the emission at pump powers below, around, and above the lasing threshold, indicating a clear linewidth narrowing at threshold

☐ Yes ☒ No

Our work studies the spatial structure properties of optical beams, Therefore, it is not relevant to the spectral measurements of the laser.

Resolution of the spectrometer used to make spectral measurements

☐ Yes ☒ No

Our work studies the spatial structure properties of optical beams, Therefore, it is not relevant to the spectral measurements of the laser.

3. Coherent emission

Measurements of the coherence and/or polarization of the emission

☐ Yes ☒ No

Our work studies the spatial structure properties of optical beams, Therefore, it is not relevant to the coherence or polarization of the laser.

4. Beam spatial profile

Image and/or measurement of the spatial shape and profile of the emission, showing a well-defined beam above threshold

☒ Yes ☐ No

In Figure 5

5. Operating conditions

Description of the laser and pumping conditions
*Continuous-wave, pulsed, temperature of operation*

☒ Yes ☐ No

In the "Methods" section

Threshold values provided as density values (e.g. W cm$^{-2}$ or J cm$^{-2}$) taking into account the area of the device

☐ Yes ☒ No

Our work studies the spatial structure properties of optical beams, Therefore, it is not relevant to the power density of the beam.

6. Alternative explanations

Reasoning as to why alternative explanations have been ruled out as responsible for the emission characteristics
*e.g. amplified spontaneous, directional scattering; modification of fluorescence spectrum by the cavity*

☐ Yes ☒ No

This study has no alternative explanations about amplified spontaneous, directional scattering; modification of fluorescence spectrum by the cavity.

7. Theoretical analysis

Theoretical analysis that ensures that the experimental values measured are realistic and reasonable
*e.g. laser threshold, linewidth, cavity gain-loss, efficiency*

☐ Yes ☒ No

This study has no theoretical analysis related to laser threshold, linewidth, cavity gain-loss, efficiency.

8. Statistics

Number of devices fabricated and tested

☐ Yes ☒ No

No device was fabricated and tested in the study.

Statistical analysis of the device performance and lifetime (time to failure)

☐ Yes ☒ No

No device was fabricated and tested in the study.



March 2018
